# A transdiagnostic approach to non-motor symptoms in Parkinson’s disease: exploring internalizing, bipolar spectrum, and impulsive compulsive behavior profiles via cluster analysis

**DOI:** 10.3389/fnagi.2026.1898498

**Published:** 2026-08-25

**Authors:** Ardıl Bayram Şahin, Mahmoud Mahmoudi, Yasemin Kadandır, İbrahim Melih Öztürkmen, Elif Sude Özaşik, Roza Gök, Muhammet Nesip Seyidoğlu, Çınar Öztosun, Özgür Öztop Çakmak, Gül Yalçın Çakmaklı, Fahriye Feriha Özer, Hale Yapici Eser

**Affiliations:** 1 Graduate School of Health Sciences, Koç University, Istanbul, Türkiye; 2 Research Center for Translational Medicine (KUTTAM), Koç University, Istanbul, Türkiye; 3 Department of Psychiatry, Faculty of Medicine, Biruni University, Istanbul, Türkiye; 4 School of Medicine, Koç University, Istanbul, Türkiye; 5 Faculty of Medicine, Hacettepe University, Ankara, Türkiye; 6 Department of Psychology, Atlas University, Istanbul, Türkiye; 7 Department of Psychiatry, School of Medicine, Rush University, Chicago, IL, United States; 8 Department of Neurology, School of Medicine, Koç University, Istanbul, Türkiye; 9 Department of Neurology, Faculty of Medicine, Hacettepe University Ankara, Türkiye; 10 Department of Neurology, Koç University Hospital, Istanbul, Türkiye; 11 Department of Psychiatry-Neuroscience, School of Medicine, Koç University, Istanbul, Türkiye

**Keywords:** bipolar spectrum, cluster analysis, impulsive-compulsive behaviors, Internalizing symptoms, non-motor symptoms, Parkinson’s disease

## Abstract

**Background:**

Parkinson’s disease (PD) is associated with diverse non-motor symptoms (NMS), including depression, anxiety, anhedonia, bipolar-spectrum features, and impulsive-compulsive behaviors (ICBs), which substantially impair quality of life. Although these domains may share dopaminergic and frontostriatal mechanisms, they are often studied separately. This study adopted a transdiagnostic, person-centered approach to identify clinically meaningful NMS profiles in PD.

**Methods:**

In this cross-sectional study, 126 patients with PD and no dementia diagnosis from two university centers completed validated assessments of ICBs, impulsivity, anhedonia, depression, anxiety, and bipolar-spectrum symptoms. K-means clustering was applied to standardized variables, and cluster validity was evaluated using multiple stability metrics.

**Results:**

The four-cluster solution demonstrated the best validity and identified Low-Symptom (44.3%), Moderate Internalizing (29.4%), Bipolar–Impulsive (16.7%), and Severe Internalizing–Impulsive (9.5%) profiles. Clusters differed significantly across depression, anxiety, anhedonia, bipolar-spectrum symptoms, ICBs, and impulsivity. The Bipolar–Impulsive group was significantly younger, whereas the Moderate Internalizing profile was older and predominantly female. The Severe Internalizing–Impulsive cluster showed the highest psychiatric burden, motor severity, disease stage, dopaminergic treatment load, and amantadine use. No significant between-cluster differences emerged in global cognitive performance.

**Conclusion:**

These findings support a transdiagnostic model in PD in which affective dysregulation and impulsivity interact with disease-related factors to form distinct neuropsychiatric phenotypes. Recognizing these profiles may facilitate earlier identification of vulnerable patients and more individualized neuropsychiatric management strategies beyond a solely dopaminergic framework.

## Introduction

Parkinson’s disease (PD) is the second most common progressive neurodegenerative disorder, characterized by the cardinal motor features of tremor, bradykinesia, and rigidity. Although motor manifestations remain central to its clinical presentation, accumulating evidence indicates that non-motor symptoms, encompass internalizing symptoms (depression, anxiety, and anhedonia), broader affective features including bipolar spectrum symptoms, as well as externalizing manifestations such as impulsive-compulsive behaviors (ICBs), are highly prevalent, affecting more than 80% of patients (Fernandes et al., 2021; Herath et al., 2024; Rodriguez-Blazquez et al., 2020). Consistent with these international findings, data from Türkiye similarly indicate a high non-motor symptom burden in PD, with more than half of patients exhibiting severe to very severe load, prominently involving mood and cognitive domains (Onder and Comoglu, 2024).

Increasing evidence suggests that non-motor symptoms in Parkinson’s disease may emerge during the prodromal phase before classical motor manifestations and constitute a heterogeneous spectrum with distinct neurobiological substrates, while also representing major determinants of health-related quality of life, often exceeding the impact of motor symptoms (Ehgoetz Martens and Shine, 2018; Heinzel et al., 2019; Kakimoto et al., 2023; Jellinger, 2025; Yapici Eser et al., 2017).

Among these, impulsive-compulsive behaviors (ICBs) have attracted particular attention due to their association with impaired inhibitory control and maladaptive reward-seeking behaviors (Carbone and Djamshidian, 2024; Gatto and Aldinio, 2019). ICBs include pathological gambling, hypersexuality, compulsive shopping, compulsive eating, punding, hobbyism, and dopamine dysregulation syndrome. Reported prevalence rates vary widely across studies, ranging from 3% to nearly 60% (Antonini et al., 2017; Parra-Díaz et al., 2020; Toś et al., 2024; Weintraub et al., 2010). Dopamine agonist use is considered the strongest risk factor; however, only a subset of treated patients develop ICBs, suggesting an important contribution of individual vulnerability factors (Grall-Bronnec et al., 2022; Molde et al., 2018; Zhang et al., 2014). Structural vulnerability factors have also been implicated; morphometric alterations in the mesocorticolimbic network, including frontal pole thinning and reduced caudate volume, have been reported in PD patients with ICBs, independent of dopaminergic medication load (Ansari et al., 2024).

Depression and anxiety are among the most prevalent internalizing symptoms in PD and meta-analytic findings indicate prevalence rates of approximately 38% for depression and 31% for anxiety disorders (Broen et al., 2016; Cong et al., 2022). These symptoms frequently co-occur with ICBs and have been linked to abnormalities in frontostriatal and mesolimbic networks thought to be involved in emotional regulation and reward processing (Edelstyn et al., 2023). Trait impulsivity and negative affect are also reported as overlapping vulnerability dimensions contributing to both affective symptoms and behavioral dysregulation in PD (Gatto and Aldinio, 2019; Averbeck et al., 2014).

Anhedonia, defined as diminished ability to experience pleasure, represents another clinically significant non-motor symptom in PD, with reported prevalence rates ranging from 7 to 45% (Loas et al., 2012). Beyond its association with depression, altered hedonic processing has also been implicated in the development of ICBs, particularly under dopaminergic treatment, where heightened reward sensitivity and impaired cognitive control may facilitate maladaptive behaviors (Houeto et al., 2016). In addition, emerging evidence suggests that bipolar spectrum features, including mood instability and subthreshold manic symptoms, may present in PD and overlap with impulsive-compulsive presentations through shared dopaminergic and reward-related mechanisms (Geelhand de Merxem et al., 2023).

Collectively, these findings support a transdiagnostic perspective in which ICBs, depression, anxiety, anhedonia, and bipolar features represent interacting dimensions of non-motor dysfunction in PD (Weintraub et al., 2022) and that these symptom domains demonstrate substantial overlap across neural, cognitive, and affective systems, suggesting broader multi-level dysfunction rather than isolated psychiatric phenomena (Edelstyn et al., 2023; Balestrino and Martinez-Martin, 2017). Nevertheless, most previous studies have examined non-motor manifestations separately, potentially overlooking their co-occurrence and shared neurobiological mechanisms in PD (Weintraub et al., 2010; Broen et al., 2016; Loas et al., 2012). A transdiagnostic perspective may better characterize this complexity by conceptualizing affective and behavioral symptoms within interconnected motivational and reward-related systems and data-driven clustering approaches can provide a more nuanced understanding of distinct neuropsychiatric profiles in PD. Based on this framework, the present study aimed to identify distinct neuropsychiatric profiles in PD using a data-driven clustering approach integrating depressive symptoms, anxiety, anhedonia, bipolar-spectrum features and impulsive-compulsive behaviors. It has been hypothesized that these symptom dimensions would form clinically meaningful subgroups characterized by differing patterns of affective dysregulation, impulsivity, and disease severity. By adopting a transdiagnostic and person-centered perspective, this study seeks to advance the understanding of non-motor heterogeneity in PD and contribute to more individualized assessment and treatment strategies beyond a solely dopaminergic framework.

## Materials and methods

### Sample

This cross-sectional study was conducted at two centers: Koç University Hospital Neurological Sciences Outpatient Clinic and Hacettepe University Neurology Outpatient Clinic. Inclusion criteria were: (i) age ≥ 18 years, (ii) diagnosis of Parkinson’s disease (Postuma et al., 2015), (iii) capacity to provide informed consent, (iv) ability to read and write in Turkish, and (v) completion of at least primary school education. Participants with atypical Parkinson’s disease or dementia diagnosis, known malignancy, other central nervous system disorders, or alcohol or substance use disorders, any medical or neurological condition that could significantly impair cognitive functioning, and with a Standardized Mini Mental State Examination (SMMSE) score lower than 19, and with a deep brain stimulation operation were excluded from the study.

For sample size calculation, a power analysis was performed using G*Power (Version 3.1.9.6) software; based on the prevalence rates of ICB and anhedonia (Erga et al., 2020; Pettorruso et al., 2014), the minimum sample size was calculated as 124 under the conditions of α = 0.05, 1-β = 0.85, and Cohen’s w = 0.316. A total of 138 patients were recruited in the study. Following data quality control procedures, 12 participants were excluded: 6 for unreliable or incomplete questionnaires, 4 for insufficient education level, and 2 for low SMMSE scores.

### Procedure

Patients were evaluated during the “on” state by the neurologist responsible for their clinical follow-up. Parkinson’s disease related clinical variables, including the Movement Disorder Society Unified Parkinson’s Disease Rating Scale Part III (MDS UPDRS III) and the Hoehn and Yahr (H&Y) stage, were assessed. Following this evaluation, cognitive assessments including SMMSE, and the Frontal Assessment Battery (FAB) were administered face-to-face. Immediately thereafter, participants completed the psychiatric scales via the Qualtrics survey system on a computer under the observation of a research assistant or with assistance provided when necessary.

### Clinical assessments

#### MDS-UPDRS III

This scale was used to assess and monitor motor symptom severity and was administered by a neurologist experienced in the field and trained in the administration of the MDS-UPDRS (Goetz et al., 2008). The Turkish validity and reliability study of the scale has been conducted and the scale is widely used in clinical research (Akbostanci et al., 2018).

#### Clinician recorded clinical variables

Clinical information including, age at Parkinson’s disease onset, disease duration, side of onset and initial presenting symptom, family history of Parkinson’s disease, and antiparkinsonian medications were recorded by the neurologist responsible for the clinical follow-up. Levodopa Equivalent Daily Dose (LEDD), a standardized measure used to quantify the total daily dopaminergic medication load by converting different antiparkinsonian drugs into a levodopa-equivalent dose, allowing comparison of treatment intensity across patients, was computed in milligrams using the following conversion ratios: standard levodopa × 1; entacapone × 0.33; tolcapone × 0.5; ropinirole × 20; rasagiline × 100; amantadine × 1; levodopa–carbidopa intestinal gel × 1.10; pramipexole × 100; rotigotine × 30; and selegiline × 10 (Tomlinson et al., 2010). Dopamine agonist LEDD (DA-LEDD), Amantadine LEDD, and MAO-B inhibitor LEDD (MAO-I LEDD) were calculated separately based on their respective medication classes.

#### H&Y

This staging scale, developed by Hoehn and Yahr (1967), was used for staging disease severity in Parkinson’s disease, with higher stages indicating more advanced disease.

#### SMMSE

This scale was developed by Folstein et al. (1975) and the Turkish standardized version of the test (SMMSE) was translated and validated by Güngen and colleagues. The SMMSE consists of five domains including orientation, immediate memory, attention and calculation, recall, and language, comprising a total of 21 items. In this study, the version standardized for educated individuals was used. In Türkiye, the cut off score for mild to moderate cognitive impairment has been established as 19/20 (Güngen et al., 2002).

#### FAB

This battery was developed by Dubois et al. (2000). The FAB consists of six subtests assessing conceptualization, mental flexibility, motor programming, sensitivity to interference, inhibitory control, and environmental autonomy, with total scores ranging from 0 to 18. The Turkish validity and reliability study of the FAB was conducted by Tuncay et al. (2013).

### Self-report measures

#### Sociodemographic data form

The sociodemographic data form used in the study was developed by the researchers. This form includes patients’ age, educational status, sex, marital status, employment status, and history of psychiatric disorders (lifetime and active), and family history of psychiatric disorders.

##### Questionnaire for Impulsive Compulsive Disorders in Parkinson’s Disease Rating Scale (QUIP-RS)

The scale was used to assess impulse control disorders and related behaviors (Weintraub et al., 2012). The QUIP RS is designed to evaluate four core impulse control disorders including pathological gambling, hypersexuality, compulsive shopping, and compulsive eating, as well as three related behaviors consisting of hobbyism, punding, and dopamine dysregulation syndrome, collectively referred to as impulsive-compulsive behaviors (ICBs). Each domain comprises four items assessing commonly reported thoughts, desires or urges, difficulty controlling behaviors, and engagement in the behavior, resulting in a total of 28 items. In the present study, QUIP-RS subscales were used to derive ICB load, defined as the total number of subtypes exceeding their respective validated cut-off thresholds (Probst et al., 2014), yielding a score ranging from 0 to 7. The Turkish validity and reliability of the QUIP-RS were established by Şahin et al. (2025)

##### Barratt Impulsiveness Scale 11 (BIS 11)

A widely used self-report measure developed by Patton et al. (1995) consists of 30 items rated on a 4-point Likert scale ranging from 1 to 4, with higher total scores indicating greater levels of impulsivity. The Turkish validity and reliability of the scale were established by Gulec et al. (2008).

##### Patient Health Questionnaire 9 (PHQ 9)

Developed by Kroenke et al. (2001), the scale consists of nine items rated on a 4-point Likert scale (0-3), yielding a total score ranging from 0 to 27. The Turkish validity and reliability of the PHQ 9 were established by Sari et al. (2016).

##### Generalized Anxiety Disorder 7 (GAD 7)

A brief clinical instrument to screen for generalized anxiety disorder (Spitzer et al., 2006), consists of seven items rated on a 4-point Likert scale (0–3), yielding a total score range of 0–21. The Turkish validity and reliability of the scale were established by Konkan et al. (2013).

##### Snaith Hamilton Pleasure Scale (SHAPS)

A self-report measure of hedonic capacity (Snaith et al., 1995), consists of 14 items rated on a 4-point Likert scale. With respect to scoring, responses of “strongly disagree”/“definitely disagree” and “disagree” are assigned a score of 1, while responses of “agree” and “strongly agree”/“definitely agree” are assigned a score of 0, resulting in a total score ranging from 0 to 14. The Turkish validity and reliability of the SHAPS were established by Yapici Eser et al. (2020).

##### Mood Disorder Questionnaire (MDQ)

A screening instrument for bipolar disorder (Hirschfeld et al., 2000), consists of 14 dichotomous yes or no items assessing lifetime manic or hypomanic symptoms. The Turkish validity and reliability of the MDQ were established by Konuk et al. (2007).

### Data analysis

#### Data preparation

Continuous variables were examined for distributional properties, outliers, and missing data. Clustering analyses were restricted to participants with complete data on the five core clustering variables: Clustering was performed on the following measures: ICBs (QUIP-RS), Anhedonia (SHAPS), Depressive symptoms (PHQ-9), Anxiety symptoms (GAD-7), Bipolar-spectrum symptoms (MDQ). ICB involvement was operationalized as ICB load, defined as the total number of ICB subtypes for which a patient exceeded the validated cut-off threshold on the QUIP-RS, yielding a score ranging from 0 to 7. This approach was preferred over the continuous total score because each ICB subtype has a distinct cut-off value, making subtype count a more clinically meaningful indicator of behavioral spectrum. Before clustering, all variables were transformed into z-scores to ensure that differences in scale did not influence the clustering solution (Gao et al., 2023). Clustering was conducted using the K-means algorithm with K-means++ initialization and Euclidean distance as the similarity metric, implemented in scikit-learn (Pedregosa et al., 2011).

The number of clusters (k) was systematically explored across a small-to-moderate range (*k* = 2–8) consistent with prior psychiatric clustering applications employing symptom-scale data (Liu et al., 2022; Mu et al., 2017). For each value of k, clustering was repeated across 100 independent runs. Within each run, k-means++ initialization was used with a high number of centroid restarts (n_init = 100) to minimize sensitivity to random initialization (Kuncheva and Kuncheva, 2006).

K-means was selected as the primary clustering algorithm because all clustering variables were continuous and standardized, making Euclidean distance an appropriate dissimilarity metric. In addition, k-means remains one of the most commonly used approaches in Parkinson’s disease subtyping research, facilitating comparison with previous studies (Hendricks and Khasawneh, 2021). To reduce dependence on a single clustering solution, multiple internal validation metrics (Silhouette coefficient, Davies–Bouldin Index, and LDA classification accuracy) were evaluated, and solution robustness was further assessed through repeated initialization and bootstrap-based stability analyses.

To evaluate reproducibility, sensitivity to random initialization was assessed by repeating K-means clustering across multiple runs and computing pairwise Jaccard similarity coefficients between resulting partitions. Robustness to sampling variability was further evaluated through bootstrap resampling, with agreement between bootstrap-derived and original partitions quantified using the Adjusted Rand Index (ARI). In addition, a high number of centroid initializations (n_init = 100) was used to reduce the likelihood of convergence to suboptimal local minima.

#### Model evaluation and selection

Candidate cluster solutions were evaluated using three complementary validation metrics: the Silhouette coefficient (Rousseeuw, 1987), which captures within-cluster cohesion and between-cluster separation; the Davies–Bouldin Index (DBI) (Davies and Bouldin, 1979), which measures average cluster separability; and Linear Discriminant Analysis (LDA)–based separability accuracy, computed by treating cluster assignments as pseudo-labels (Fisher, 1936). LDA was applied as a diagnostic tool to quantify the linear separability of derived clusters in multivariate space, rather than as a supervised classification model.

As no single metric provides a definitive criterion for determining the optimal number of clusters, validation indices were interpreted jointly. To enable comparison across candidate solutions, a composite performance score was computed by combining normalized DBI, Silhouette values, and LDA accuracy, together with their stability across repeated runs, quantified as the standard deviation of each metric. In addition, k-means inertia (i.e., within-cluster sum of squared distances) was examined using the elbow method to identify points of diminishing returns with increasing numbers of clusters (k) (Liu et al., 2022).

The final model was selected by balancing quantitative criteria with clinical interpretability, prioritizing solutions that demonstrated both statistical robustness and clinically meaningful differentiation across clusters.

#### Between-cluster comparisons

Following identification of the final cluster solution, clusters were compared on clinical and demographic variables (age, sex, marital status, psychiatric history), PD clinical characteristics (UPDRS-III, H&Y stage, disease duration, family history), PD Treatment characteristics (Treatment usage, Levodopa usage, DA usage, Amantadine usage, MAO-I usage, and LEDD values), PD non-motor symptoms (ICBs, anhedonia, depression, anxiety, and bipolar spectrum features), cognitive features (SMMSE, FAB), and impulsivity (BIS-11). Due to non-normal distributions, continuous variables were analyzed using Kruskal–Wallis tests, with *post hoc* pairwise comparisons performed using Dunn’s test with Bonferroni correction when overall group differences were significant. Categorical variables were compared using chi-square tests, with Fisher’s exact test applied when expected cell frequencies were below five. For categorical variables, Bonferroni correction was also applied for *post hoc* pairwise comparisons across clusters.

To examine whether cluster membership was associated with demographic and disease-related characteristics, an exploratory multinomial logistic regression analysis was performed using cluster membership as the dependent variable, with the Low-Symptom profile as the reference category. Age, sex, MDS-UPDRS Part III score, and total LEDD were entered as predictors. Psychiatric symptom measures used to derive the clusters (PHQ-9, GAD-7, SHAPS, MDQ, and QUIP-RS) were not included in the model to avoid circularity. Odds ratios (ORs) and 95% confidence intervals (CIs) were calculated for all predictors.

All analyses were conducted using either Python (scikit-learn library) and IBM SPSS Statistics version 28. A two-tailed *p* < 0.05 was considered statistically significant.

## Results

### Sample characteristics

Of the 126 participants who met the validity and inclusion criteria the mean age was 64.67 ± 10.65; 53.2% were male and 73.8% were married. Most participants were retired or unemployed (77%) and had at least a high school education (61.9%).

In terms of PD treatment, most participants were receiving antiparkinsonian medications (93.7%), with 74.6% using levodopa, 55.6% using dopamine agonists, 32.5% using MAO inhibitors, 19.8% using Amantadine; the mean Total LEDD was 499.76 ± 350.19 mg.

Based on clinician-based evaluation, mean disease duration was 63.33 ± 52.33 months, MDS-UPDRS III score was 18.78 ± 11.24, and the Hoehn and Yahr stage was 1.73 ± 0.71 (Only 1 patient was stage 4).

Additional sociodemographic, clinical, and treatment-related characteristics of the sample are provided in Table 1.

**TABLE 1:** Socio-demographic and clinical characteristics of the sample (*n* = 126).

Socio-demographic characteristics	Min–max	Mean ( ± SD) or *n* (%)
Age	34–87	64.67 ± 10.65
Sex		
Female		59 (46.8)
Male		67 (53.2)
Marital status		
Married		93 (73.8)
Non-married/divorced		33 (26.2)
Employment		
Active worker		29 (23)
Retired/unemployed		97 (77)
Education		48 (38.1)
Primary/middle school		78 (61.9)
High school or above		
**Clinical characteristics**		**Mean ( ±** **SD) or n (%)**
PD duration (month)	1–276	63.33 ± 52.33
MDS-UPDRS III score	2–55	18.78 ± 11.24
Hoehn and Yahr stage	1–4	1.73 ± 0.71
Family history of PD		25 (19.8)
Psychiatric disorder (active)		25 (19.8)
Psychiatric disorder (lifetime)		35 (27.8)
**Treatment characteristics**		**Mean ( ±** **SD) or n (%)**
Any treatment usage		118 (93.7)
Levodopa usage		94 (74.6)
DA usage		70 (55.6)
MAO inhibitor usage		41 (32.5)
Amantadine usage		25 (19.8)
Total LEDD	0–1500	499.76 ± 350.19
Levodopa LEDD	0–1200	304.33 ± 281.72
DA LEDD	0–467.1	114.88 ± 139.34
MAO inhibitor LEDD	0–300	33.73 ± 52.09
Amantadine LEDD	0–500	47.62 ± 105.80
**Psychometric measures**		**Mean ±** **SD**
PHQ-9	0–19	7.41 ± 4.36
GAD-7	0–20	5.20 ± 4.53
SHAPS	0–11	1.31 ± 1.80
MDQ	0–12	2.79 ± 2.87
BIS-11	37–86	57.71 ± 10.04
SMMSE	19–30	26.63 ± 2.88
FAB	5–18	14.05 ± 3.18

PD, Parkinson’s Disease; MDS-UPDRS III, Movement Disorder Society-Unified Parkinson’s disease rating scale part III; DA, Dopamine Agonist; MAO, Monoamine oxidase; LEDD, Levodopa equivalent daily dose; PHQ-9, Patient Health Questionnaire-9; GAD-7, General Anxiety Disorder-7; SHAPS, Snaith-Hamilton Pleasure Scale; MDQ, Mood Disorder Questionnaire; BIS-11, Barratt Impulsivity Scale; SMMSE, Standardized Mini-Mental State Examination; FAB, Frontal Assessment Battery; SD, Standard Deviation.

Regarding the clinical profile of ICBs, overall, 46% of the sample met criteria for at least one ICB subtype based on QUIP-RS cut-off scores (Probst et al., 2014). The most common subtypes were punding (32.5%), DDS (29.4%), and compulsive eating (26.2%) (Supplementary Tables 1A–C).

### Evaluation of candidate cluster solutions

**FIGURE 1:**
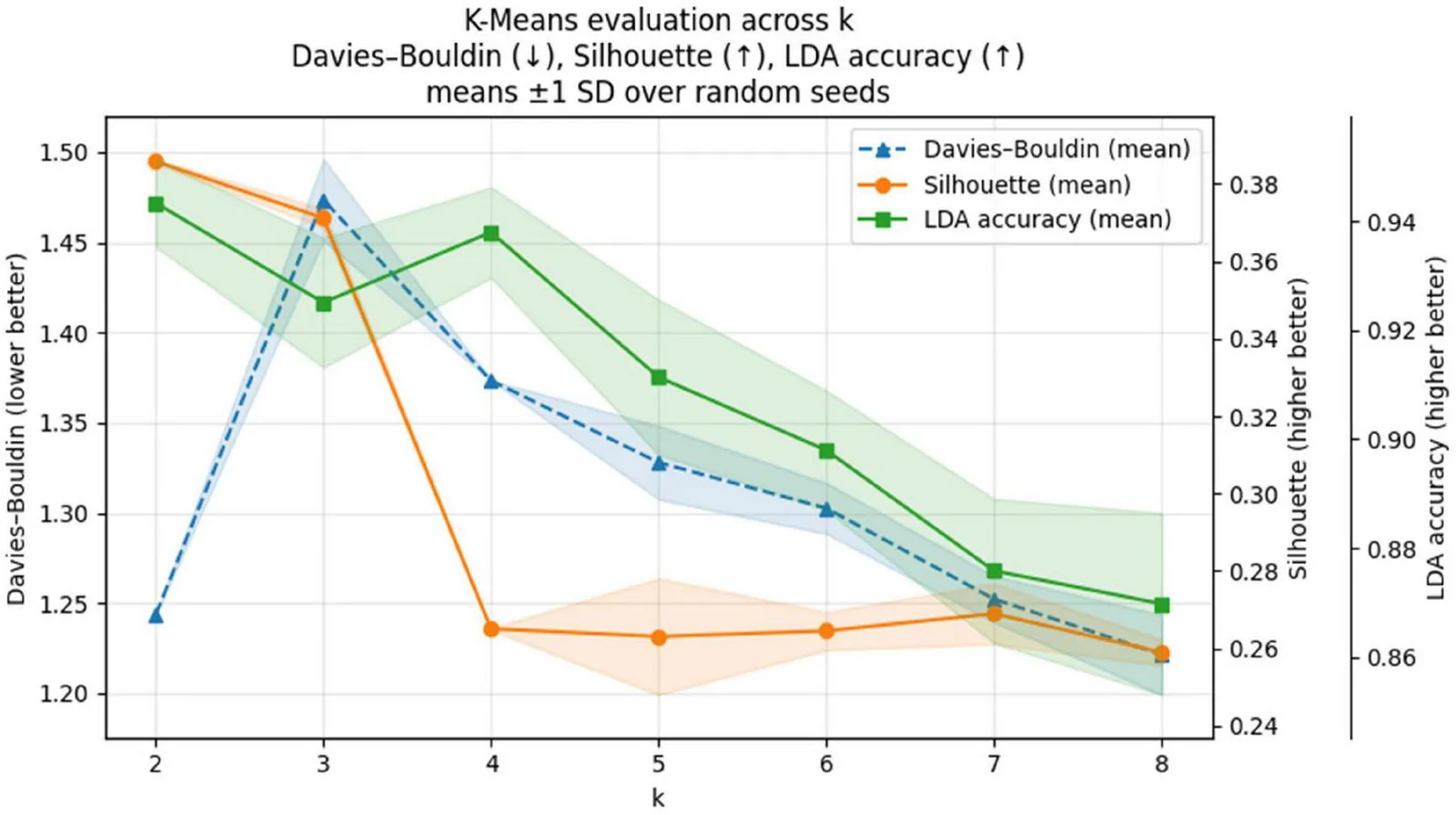
Evaluation of clustering solutions across values of k. Mean Davies–Bouldin Index (DBI), Silhouette coefficient, and Linear Discriminant Analysis (LDA) classification accuracy were evaluated for cluster solutions ranging from *k* = 2 to *k* = 8. Reported values reflect the mean ± standard deviation across multiple random initializations. Lower DBI values indicate better cluster compactness and separation, whereas higher Silhouette coefficients and LDA accuracy indicate improved clustering quality and separability.

Clustering solutions were systematically evaluated across values of k ranging from 2 to 8 using complementary internal and external validation indices. Mean Davies–Bouldin Index (DBI), Silhouette coefficients, and Linear Discriminant Analysis (LDA)–based separability accuracy across repeated random initializations are presented in Figure 1.

**FIGURE 2:**
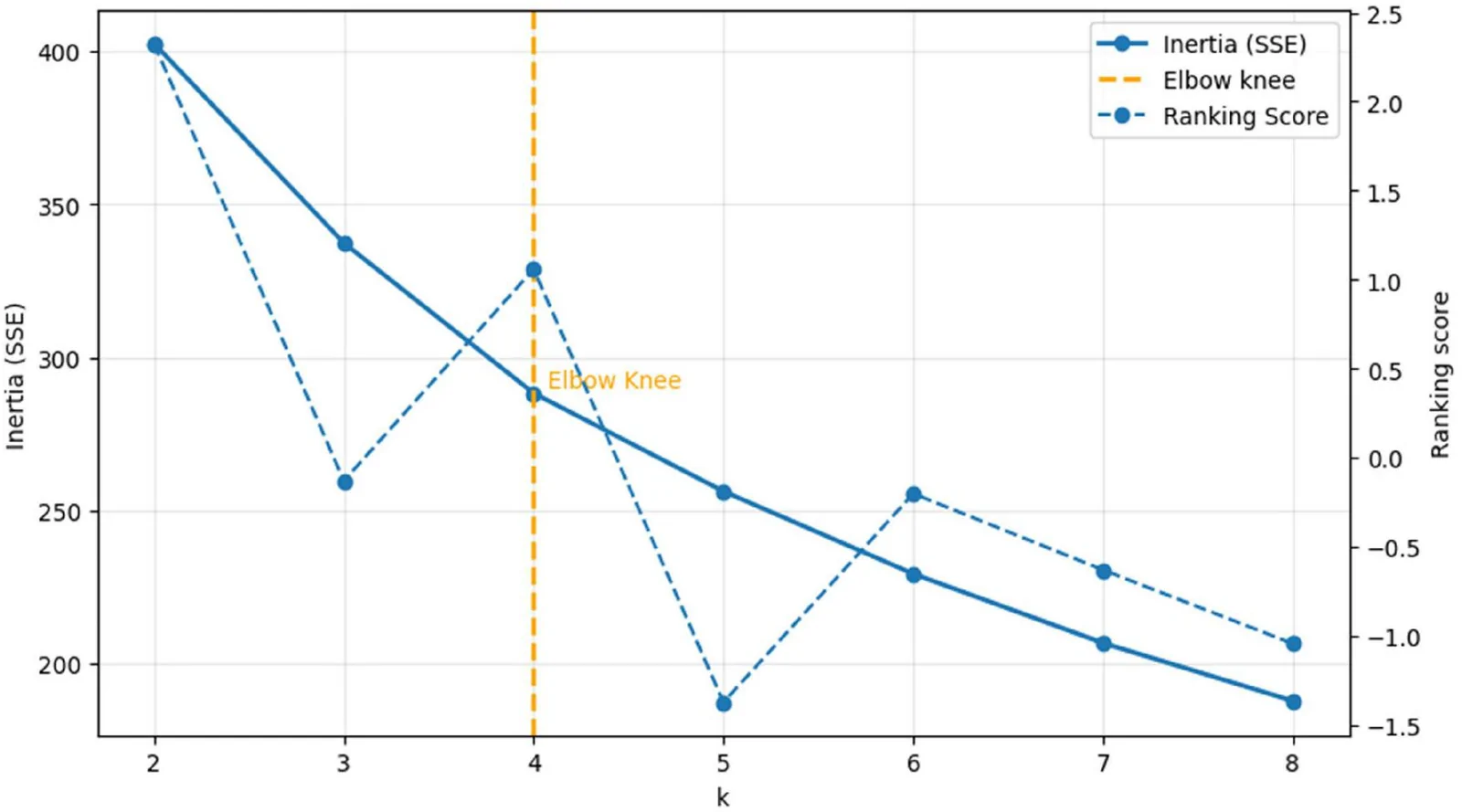
Inertia and composite ranking score across candidate cluster solutions. K-means inertia (sum of squared errors) and composite ranking scores are plotted across different values of k. The dashed vertical line marks the elbow region identified from the inertia curve. Composite ranking scores combine normalized clustering performance and stability metrics across repeated runs, with higher values indicating more optimal solutions.

To integrate clustering quality and robustness, a composite ranking score combining normalized performance (DBI, Silhouette coefficient, and LDA accuracy) and stability across runs was computed and examined alongside k-means inertia using the elbow method in Figure 2.

Evaluation using inertia (SSE) and the composite ranking score identified *k* = 4 as the primary candidate, supported by a clear elbow point. While inertia continued to decrease beyond *k* = 4, improvements became more gradual, consistent with diminishing returns in compactness. Notably, *k* = 3 coincided with a local peak in the Davies-Bouldin index and a drop in LDA accuracy relative to neighboring solutions, indicating poorer cluster separation at this value. Although no single validation metric uniformly favored one solution, *k* = 4 showed the most favorable overall balance across inertia, composite ranking, LDA-based separability, and interpretability. A secondary stabilization was observed around *k* = 6, suggesting that this solution may represent a higher-resolution partition of the data. Taken together, these findings supported *k* = 4 as the most parsimonious solution, with *k* = 6 retained as a higher-resolution alternative allowing for more fine-grained examination of subgroup structure.

### Selection of the final cluster solution

**FIGURE 3:**
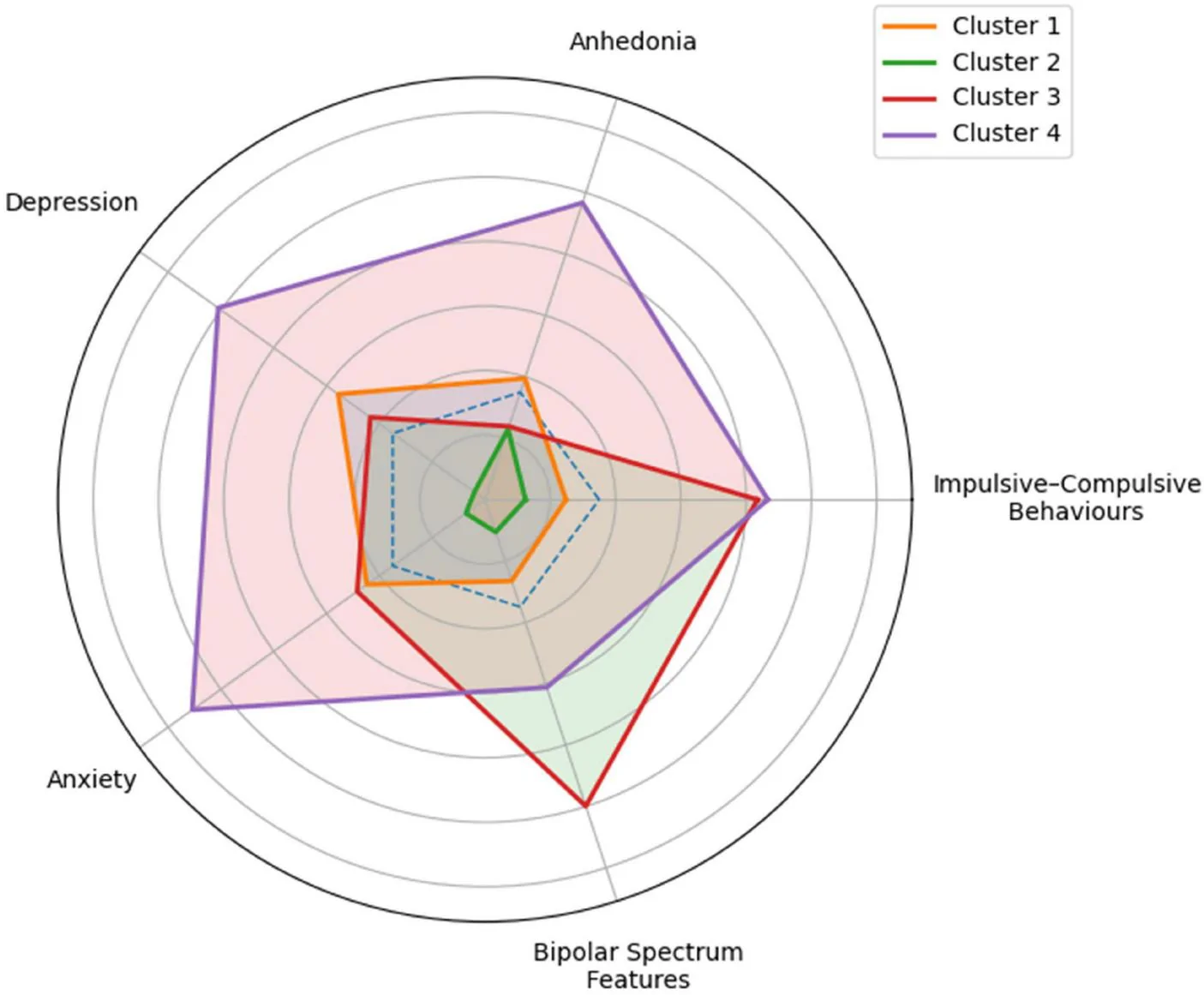
Radar plot of four-cluster non-motor profiles. Radar plot depicting standardized mean scores (z-scores) across key domains for the four identified clusters: moderate internalizing, low-symptom (normative), bipolar–impulsive, and severe internalizing–impulsive profiles. Variables include depression (PHQ-9), anxiety (GAD-7), anhedonia (SHAPS), bipolar spectrum features (MDQ), and impulse–compulsive behaviors (QUIP-RS). The profiles delineate distinct affective pathways underlying impulse–compulsive behaviors in Parkinson’s disease, with each cluster characterized by a unique configuration of internalizing symptom burden, bipolar spectrum features, and impulsivity-related manifestations.

Given the support for *k* = 4 from the elbow method and composite ranking score, together with acceptable LDA-based separability and improved DBI relative to the poorer *k* = 3 solution, final model selection was guided by interpretability and parsimony. Visual inspection of cluster profiles revealed that the four-cluster solution yielded distinct profiles across ICBs, anhedonia, depressive, anxiety, and bipolar spectrum symptoms (Figure 3). In contrast, the six-cluster solution introduced profiles with highly similar geometries differing primarily in magnitude rather than pattern, indicating limited additional differentiation (Supplementary Figure 1 and Supplementary Table 2). Accordingly, the four-cluster solution was selected as the final model.

Stability analyses indicated consistent convergence of the four-cluster solution across repeated optimization procedures. Mean pairwise Jaccard similarity was 0.64 (SD = 0.13) under single-initialization conditions and increased to ≈ 1.00 under extensive optimization (n_init = 100). Bootstrap resampling further demonstrated reproducible structure under sampling perturbation (mean ARI = 0.63). A comprehensive overview of stability metrics and clustering behavior across candidate solutions is provided in Supplementary Figures 2, 3.

### Description of clinical profiles

#### Between-cluster comparisons

##### Socio-demographic characteristics

Between-cluster differences in socio-demographic characteristics are presented in Table 2. Clusters differed significantly in terms of age [*H*(3) = 13.49, *p* = 0.004] and sex distribution [χ^2^(3) = 9.43, *p* = 0.024]. *Post-hoc* analyses revealed that Cluster 3 was significantly younger than Cluster 1 (*p* = 0.007), Cluster 2 (*p* = 0.021), and Cluster 4 (*p* = 0.044). Cluster 1 had the highest mean age, whereas Cluster 3 had the lowest. The proportion of female participants was highest in Cluster 1 (67.6%). No significant between-cluster differences were observed for marital status, employment status, education level, or monthly income (all *p* > 0.05)

**TABLE 2:** Demographic and psychiatric characteristics across clusters.

	Cluster 1 (*n* = 37)	Cluster 2 (*n* = 56)	Cluster 3 (*n* = 21)	Cluster 4 (*n* = 12)	Kruskal–Wallis test			Dunn’s test	
Variable	Mean ± SD	Mean ± SD	Mean ± SD	Mean ± SD	Value (H)	df	*P*	Pairwise comparisons	Adj. Sig
Age	68.27 ± 10.39	65.57 ± 9.91	58.57 ± 11.14	60.08 ± 8.82	**13.084**	3	0.004*	Cluster 1- Cluster 2	1.000
								Cluster 1- Cluster 3	0.009*
								Cluster 1- Cluster 4	0.101
								Cluster 2- Cluster 3	0.088
								Cluster 2- Cluster 4	0.499
								Cluster 3- Cluster 4	1.000
	**Cluster 1 (*n* = 37)**	**Cluster 2 (*n* = 56)**	**Cluster 3 (*n* = 21)**	**Cluster 4 (*n* = 12)**	**Chi-square test**			**Chi-square test**	
**Variable**	**n (%)**	**n (%)**	**n (%)**	**n (%)**	**Value (χ^2^)**	**df**	***P***	**Pairwise comparisons**	***P***
Female sex	25 (67.6)	20 (35.7)	9 (42.9)	5 (41.7)	9.431	3	0.024*	Cluster 1- Cluster 2	0.003*
								Cluster 1- Cluster 3	0.066
								Cluster 1- Cluster 4	0.173^a^
								Cluster 2- Cluster 3	0.565
								Cluster 2- Cluster 4	0.748^a^
								Cluster 3- Cluster 4	0.947
Education Primary/middle	20 (54.1)	19 (33.9)	3 (14.3)	6 (50)	10.177	3	0.017*	Cluster 1- Cluster 2	0.054
								Cluster 1- Cluster 3	0.003*
school High school or above	17 (45.9)	37 (66.1)	18 (85.7)	6 (50)				Cluster 1- Cluster 4	0.807
								Cluster 2- Cluster 3	0.089
								Cluster 2- Cluster 4	0.295
								Cluster 3- Cluster 4	0.027
Psychiatric disorder (active)	8 (21.6)	4 (7.1)	5 (23.8)	8 (66.7)	22.503	3	< 0.001**	Cluster 1- Cluster 2	0.058^a^
								Cluster 1- Cluster 3	1.000^a^
								Cluster 1- Cluster 4	0.010^a^
								Cluster 2- Cluster 3	0.057^a^
								Cluster 2- Cluster 4	< 0.001^a^ *
								Cluster 3- Cluster 4	0.027^a^
Psychiatric disorder (lifetime)	13 (35.1)	8 (33.3)	7 (28.6)	7 (58.3)	11.987	3	0.007*	Cluster 1- Cluster 2	0.019
								Cluster 1- Cluster 3	0.890
								Cluster 1- Cluster 4	0.189^a^
								Cluster 2- Cluster 3	0.102^a^
								Cluster 2- Cluster 4	0.003^a^ *
								Cluster 3- Cluster 4	0.162
Family history of PD	6 (16.2)	12 (21.4)	4 (19)	3 (25)	2.517	3	0.896	Pairwise comparisons not performed	

Data are presented as mean (SD) for continuous variables and n (%) for categorical variables. *P*-values were calculated using the Kruskal–Wallis test for continuous variables and the χ^2^ test for categorical variables. Pairwise comparisons for continuous variables were performed using Dunn–Bonferroni *post-hoc* tests, with adjusted *p*-values (Adj. Sig.) reflecting Bonferroni correction for multiple comparisons (*p < 0.05, **p < 0.001). Pairwise comparisons were performed using Chi-Square test with Bonferroni correction (*p < 0.008).

*^a^*Fisher’s Exact Test; SD, standard deviation.

##### Core clinical dimensions

Significant between-cluster differences were observed across all clustering variables. Kruskal–Wallis analyses revealed robust group differences in depressive symptoms [PHQ-9; *H*(3) = 78.54, *p* < 0.001], anxiety symptoms [GAD-7; *H*(3) = 69.70, *p* < 0.001], anhedonia [SHAPS; *H*(3) = 24.52, *p* < 0.001], bipolar features [MDQ; *H*(3) = 70.923, *p* < 0.001], and impulsive-compulsive behaviors [QUIP-RS; *H*(3) = 65.101, *p* < 0.001].

Between-cluster differences in core clinical dimensions are presented in Table 3. *Post hoc* analyses indicated that Cluster 2 consistently exhibited the lowest levels across all measures. In contrast, Cluster 4 showed the highest levels of depressive symptoms, anxiety, and anhedonia, along with elevated ICBs. Cluster 3 was distinguished by markedly higher bipolar features and elevated ICBs, whereas Cluster 1 showed intermediate levels of depressive and anxiety symptoms, significantly higher than the low-symptom profile but not significantly different from cluster 3 on these measures. Notably, ICBs were similarly elevated in Clusters 3 and 4. *Post hoc* analyses for BIS-11 indicated a similar pattern to ICBs, with significantly higher impulsivity in Clusters 3 and 4 compared to Cluster 2 (both *p* < 0.001), and higher levels in Cluster 3 and Cluster 4 compared to Cluster 1 (respectively, *p* = 0.016 and *p* = 0.002), while no differences were observed between Clusters 3 and 4 (*p* = 1.000).

**TABLE 3:** Comparisons of depression, anxiety, anhedonia, mood instability and ICBs across the clusters.

	Cluster 1 (*n* = 37)	Cluster 2 (*n* = 56)	Cluster 3 (*n* = 21)	Cluster 4 (*n* = 12)	Kruskal–Wallis test			Dunn’s test	
Variables	Mean ± SD	Mean ± SD	Mean ± SD	Mean ± SD	Test Statistic (H)	df	*P*	Pairwise comparisons	Adj. Sig.
PHQ-9	9.65 ± 2.70	4.05 ± 1.77	8.33 ± 4.10	14.58 ± 3.68	78.542	3	< 0.001**	Cluster 1- Cluster 2	< 0.001**
								Cluster 1- Cluster 3	0.749
								Cluster 1- Cluster 4	0.306
								Cluster 2- Cluster 3	< 0.001**
								Cluster 2- Cluster 4	< 0.001**
								Cluster 3- Cluster 4	0.019*
GAD-7	6.30 ± 3.78	2.07 ± 1.61	6.71 ± 3.05	13.75 ± 4.27	69.698	3	< 0.001**	Cluster 1- Cluster 2	< 0.001**
								Cluster 1- Cluster 3	1.000
								Cluster 1- Cluster 4	0.012*
								Cluster 2- Cluster 3	< 0.001**
								Cluster 2- Cluster 4	< 0.001**
								Cluster 3- Cluster 4	0.133
SHAPS	1.51 ± 1.43	0.77 ± 1.13	0.81 ± 1.21	4.08 ± 3.29	24.52	3	< 0.001**	Cluster 1- Cluster 2	0.044*
								Cluster 1- Cluster 3	0.192
								Cluster 1- Cluster 4	0.070
								Cluster 2- Cluster 3	1.000
								Cluster 2- Cluster 4	< 0.001**
								Cluster 3- Cluster 4	< 0.001**
MDQ	2.19 ± 1.65	1.05 ± 1.42	7.43 ± 2.14	4.67 ± 2.15	70.923	3	< 0.001**	Cluster 1- Cluster 2	0.015*
								Cluster 1- Cluster 3	< 0.001**
								Cluster 1- Cluster 4	< 0.001**
								Cluster 2- Cluster 3	0.076
								Cluster 2- Cluster 4	< 0.001**
								Cluster 3- Cluster 4	0.770
QUIP-RS	0.89 ± 1.20	0.29 ± 0.76	3.76 ± 2.07	3.92 ± 1.93	65.101	3	< 0.001**	Cluster 1- Cluster 2	0.148
								Cluster 1- Cluster 3	< 0.001**
								Cluster 1- Cluster 4	< 0.001**
								Cluster 2- Cluster 3	< 0.001**
								Cluster 2- Cluster 4	< 0.001**
								Cluster 3- Cluster 4	1.000

Values are presented as test statistics from Kruskal–Wallis analyses. Pairwise comparisons were performed using Dunn–Bonferroni *post-hoc* tests when the overall test was significant. Adjusted *p*-values (Adj. Sig.) reflect Bonferroni correction for multiple comparisons. PHQ-9, Patient Health Questionnaire-9; GAD-7, General Anxiety Disorder-7; SHAPS, Snaith-Hamilton Pleasure Scale; MDQ, Mood Disorder Questionnaire; QUIP-RS, Questionnaire for Impulsive-Compulsive Disorders in Parkinson’s Disease–Rating Scale; SD, standard deviation. *p < 0.05, **p < 0.001.

Based on these sociodemographic and clinical properties, the four identified clinical profiles were characterized and named as follows:

*Cluster 1 Moderate Internalizing Profile:* Patients in this cluster exhibited moderate levels of depressive symptoms and anxiety, accompanied by mildly elevated anhedonia, while impulsivity and bipolar spectrum features remained relatively low.

*Cluster 2 Low-Symptom Profile:* This cluster was characterized by consistently low levels across all clinical dimensions, including depressive symptoms, anxiety, anhedonia, bipolar features, and ICBs, representing a relatively asymptomatic or normative clinical profile.

*Cluster 3 Bipolar-Impulsive Profile:* Patients in this cluster demonstrated markedly elevated bipolar features and high levels of ICBs, alongside moderate levels of depressive symptoms and anxiety and low anhedonia. This pattern suggests an impulsivity-driven profile closely associated with bipolar features rather than internalizing burden.

*Cluster 4 Severe Internalizing-Impulsive Profile:* This cluster exhibited high levels of depressive symptoms, anxiety, and anhedonia, together with elevated ICBs and moderate bipolar features. The co-occurrence of severe internalizing symptoms and impulsivity indicates a highly burdened clinical profile representing the most symptomatic subgroup (Figure 3).

##### Clinical characteristics and medication use

Significant differences were observed across clusters in the prevalence of current (*p* < 0.001) and lifetime psychiatric disorders (*p* = 0.007), whereas no differences were found for family history of Parkinson’s disease (*p* = 0.896). Both current and lifetime psychiatric disorders were more frequent in Cluster 4 (severe internalizing-impulsive profile). Regarding medication use, clusters did not differ significantly in terms of overall treatment use (*p* = 0.535), levodopa use (*p* = 0.517), dopamine agonist use (*p* = 0.724), or MAO inhibitor use (*p* = 0.602). In contrast, amantadine use differed significantly across clusters (*p* = 0.002), with higher prevalence in Clusters 3 (Bipolar-impulsive profile) and 4 (severe internalizing-impulsive profile). There was no difference between the clusters in terms of the use of levodopa alone, dopamine agonists alone or levodopa combined with dopamine agonists (*p* = 0.167). Between-cluster differences in clinical characteristics and medication use are presented in Supplementary Table 3

##### Cluster differences in motor severity, disease stage, and medication load

Significant between-cluster differences were observed in motor severity [MDS–UPDRS III; *H*(3) = 9.82, *p* = 0.020], disease stage [Hoehn and Yahr; *H*(3) = 17.60, *p* < 0.001], total dopaminergic medication load [total LEDD; *H*(3) = 11.50, *p* = 0.009], and Amantadine load [Amantadine LEDD; *H*(3) = 15.36, *p* = 0.002]. *Post hoc* analyses indicated that these differences were primarily driven by higher values in Cluster 4. No significant differences were observed for other medication variables (all *p* > 0.05).

Between-cluster differences in motor severity, disease stage, and medication load are presented in Table 4. There were no significant differences observed in total cognitive scores. The comparison of the SMMSE and FAB total scores and subdomain scores across groups is presented in Table 5.

**TABLE 4:** Comparison of motor severity, disease stage, and dopaminergic medication load across clusters.

	Cluster 1 (*n* = 37)	Cluster 2 (*n* = 56)	Cluster 3 (*n* = 21)	Cluster 4 (*n* = 12)	Kruskal–Wallis test			Dunn’s test	
Variables	Mean ± SD	Mean ± SD	Mean ± SD	Mean ± SD	Test statistic (H)	df	P	Pairwise comparisons	Adj. Sig.
MDS-UPDRS III	17.97 ± 10.67	17.98 ± 11.69	16.76 ± 8.97	28.50 ± 10.88	9.819	3	0.020*	Cluster 1- Cluster 2	1.000
								Cluster 1- Cluster 3	1.000
								Cluster 1- Cluster 4	0.033*
								Cluster 2- Cluster 3	1.000
								Cluster 2- Cluster 4	0.017*
								Cluster 3- Cluster 4	0.037*
Hoehn and Yahr stage	1.81 ± 0.70	1.61 ± 0.62	1.48 ± 0.68	2.50 ± 0.67	17.601	3	< 0.001**	Cluster 1- Cluster 2	1.000
								Cluster 1- Cluster 3	0.396
								Cluster 1- Cluster 4	0.038*
								Cluster 2- Cluster 3	1.000
								Cluster 2- Cluster 4	< 0.001**
								Cluster 3- Cluster 4	< 0.001**
Total LEDD	475.61 ± 305.38	445.66 ± 369.61	518.69 ± 324.94	793.54 ± 318.72	11.499	3	0.009*	Cluster 1- Cluster 2	1.000
								Cluster 1- Cluster 3	1.000
								Cluster 1- Cluster 4	0.038*
								Cluster 2- Cluster 3	1.000
								Cluster 2- Cluster 4	0.005*
								Cluster 3- Cluster 4	0.198
Amantadine LEDD	22.97 ± 60.78	32.14 ± 99.28	80.95 ± 124.98	137.50 ± 152.44	15.357	3	0.002*	Cluster 1- Cluster 2	1.000
								Cluster 1- Cluster 3	0.153
								Cluster 1- Cluster 4	0.017*
								Cluster 2- Cluster 3	0.080
								Cluster 2- Cluster 4	0.008*
								Cluster 3- Cluster 4	1.000
Levodopa LEDD	297.30 ± 275.87	290.54 ± 301.94	250.60 ± 229.35	484.38 ± 243.62	6.755	3	0.080	Pairwise comparisons not performed	
DA LEDD	118.85 ± 140.03	89.06 ± 119.46	153.81 ± 156.19	155.00 ± 180.73	2.751	3	0.432	Pairwise comparisons not performed	
MAO-I LEDD	36.49 ± 48.09	35.71 ± 58.55	33.33 ± 48.31	16.67 ± 38.93	1.690	3	0.639	Pairwise comparisons not performed	
Disease duration (months)	53.11 ± 43.21	61.75 ± 46.49	70.05 ± 62.01	90.50 ± 77.20	2.407	3	0.492	Pairwise comparisons not performed	
BIS-11	56.03 ± 7.45	53.46 ± 7.33	64.91 ± 11.44	70.08 ± 9.46	36.187	3	< 0.001**	Cluster 1- Cluster 2	0.734
								Cluster 1- Cluster 3	0.016*
								Cluster 1- Cluster 4	0.002*
								Cluster 2- Cluster 3	< 0.001**
								Cluster 2- Cluster 4	< 0.001**
								Cluster 3- Cluster 4	1.000

Values are presented as test statistics from Kruskal–Wallis analyses. Pairwise comparisons were performed using Dunn–Bonferroni *post-hoc* tests when the overall test was significant. Adjusted *p*-values (Adj. Sig.) reflect Bonferroni correction for multiple comparisons. MDS-UPDRS III, Movement Disorder Society-Unified Parkinson’s disease rating scale part III; LEDD, Levodopa equivalent daily dose; DA, Dopamine Agonist; MAO, Monoamine oxidase; BIS-11, Barratt Impulsivity Scale. *p < 0.05 **p < 0.001.

**TABLE 5:** Comparison of total and subscales of SMMSE and FAB scores across clusters.

	Cluster 1 (*n* = 37)	Cluster 2 (*n* = 56)	Cluster 3 (*n* = 21)	Cluster 4 (*n* = 12)	Kruskal–Wallis test			Dunn’s test	
Variables	Mean ± SD	Mean ± SD	Mean ± SD	Mean ± SD	Test statistic (H)	df	*P*	Pairwise comparisons	Adj. Sig.
SMMSE	26.32 ± 2.75	26.98 ± 2.83	26.81 ± 2.60	25.67 ± 3.87	2.182	3	0.535	Pairwise comparisons not performed	
Orientation	9.11 ± 1.10	9.57 ± 0.78	9.14 ± 0.96	8.58 ± 1.24	12.911	3	0.005*	Cluster 1- Cluster 2	0.124
								Cluster 1- Cluster 3	1.000
								Cluster 1- Cluster 4	0.779
								Cluster 2- Cluster 3	0.309
								Cluster 2- Cluster 4	0.011*
								Cluster 3- Cluster 4	1.000
Registration	3.00 ± 0.00	3.00 ± 0.00	3.00 ± 0.00	3.00 ± 0.00	0.000	3	1.000	Pairwise comparisons not performed	
Attention	3.89 ± 1.66	3.98 ± 1.48	4 ± 1.34	4 .00 ± 1.21	0.260	3	0.967	Pairwise comparisons not performed	
Recall	2.05 ± 0.88	2.32 ± 0.77	2.29 ± 0.90	2.25 ± 1.06	2.382	3	0.497	Pairwise comparisons not performed	
Language	8.27 ± 0.99	8.11 ± 1.07	8.38 ± 0.97	7.83 ± 1.75	1.472	3	0.689	Pairwise comparisons not performed	
FAB	13.65 ± 3.51	14.18 ± 2.74	15.19 ± 2.54	12.67 ± 4.48	4.006	3	0.261	Pairwise comparisons not performed	
Conceptualization	1.73 ± 0.96	2.02 ± 0.80	2.24 ± 0.83	1.50 ± 1.00	7.503	3	0.057	Pairwise comparisons not performed	
Mental flexibility	2.03 ± 1.01	2.07 ± 0.87	2.48 ± 0.81	1.75 ± 1.29	4.654	3	0.199	Pairwise comparisons not performed	
Motor programming	2.54 ± 0.77	2.48 ± 0.69	2.67 ± 0.58	2.42 ± 1.00	1.431	3	0.698	Pairwise comparisons not performed	
Sensitivity to interference	2.38 ± 0.98	2.41 ± 0.83	2.76 ± 0.63	1.92 ± 1.31	5.882	3	0.118	Pairwise comparisons not performed	
Inhibitory control	1.97 ± 1.09	2.21 ± 1.06	2.14 ± 0.96	2.08 ± 1.17	1.583	3	0.663	Pairwise comparisons not performed	
Environmental autonomy	3.00 ± 0.00	2.98 ± 0.13	2.90 ± 0.30	3.00 ± 0.00	5.843	3	0.119	Pairwise comparisons not performed	

Values are presented as test statistics from Kruskal–Wallis analyses. Pairwise comparisons were performed using Dunn–Bonferroni *post-hoc* tests when the overall test was significant. Adjusted p-values (Adj. Sig.) reflect Bonferroni correction for multiple comparisons. SMMSE, Standardized Mini-Mental State Examination; FAB, Frontal Assessment Battery; SD, standard deviation. *p < 0.05.

##### Covariate-adjusted analysis of cluster membership

To assess whether cluster membership was explained by demographic and disease-related characteristics, a multinomial logistic regression was performed using the Low-Symptom profile as the reference category. The overall model was significant [χ^2^(12) = 36.26, *p* < 0.001] and explained a modest proportion of the variance in cluster membership (Nagelkerke *R*^2^ = 0.28). Female sex was independently associated with the Moderate Internalizing profile (OR = 3.61, 95% CI 1.49–8.78, *p* = 0.005), whereas younger age was associated with the Bipolar-Impulsive profile (OR = 0.94, 95% CI 0.89–0.99, *p* = 0.014). For the Severe Internalizing-Impulsive profile, higher dopaminergic treatment load (LEDD) was independently associated with cluster membership (OR = 1.002, 95% CI 1.000–1.004, *p* = 0.043), while motor severity showed a trend-level association (MDS-UPDRS III, *p* = 0.054). Overall, these findings suggest that demographic and disease-related variables contribute only partially to cluster membership and do not fully account for the observed neuropsychiatric profiles.

## Discussion

### Transdiagnostic structure of non-motor symptoms

In this study, patients with PD without dementia were included, distinct clusters of patients were identified based on depressive symptoms, anxiety, anhedonia, bipolar features, and ICBs. The four-cluster solution was selected over the six-cluster alternative based on convergent evidence from multiple validation metrics, including the elbow point in the inertia curve, favorable Davies-Bouldin index and Silhouette coefficient values, and high LDA classification accuracy. Although the six-cluster solution demonstrated a secondary stabilization in validation indices, visual inspection of cluster profiles revealed that the additional resolution did not yield qualitatively distinct new profiles. Specifically, the Moderate Internalizing Profile was subdivided into two clusters with highly similar affective geometries differing primarily in overall symptom severity rather than profile pattern, offering limited additional clinical differentiation. Similarly, the Bipolar-Impulsive profile was split into an ICB-dominant and a bipolar-dominant subgroup; while this subdivision may reflect a meaningful distinction at a finer resolution, the resulting subgroup sizes rendered these profiles statistically unreliable and clinically difficult to distinguish. The structural integrity of the Low-Symptom and Severe Internalizing-Impulsive profiles was preserved across both solutions, further supporting their robustness as core neuropsychiatric subtypes in this sample. The four-cluster solution therefore represented the most parsimonious balance between statistical validity, stability, and clinical interpretability, capturing the clinically meaningful heterogeneity of ICB profiles in PD without overfitting to sample-specific variance.

Although the four-cluster solution demonstrated near-perfect convergence under extensive optimization (n_init = 100) and moderate-to-substantial bootstrap reproducibility (mean ARI = 0.63), stability was comparatively lower under single-initialization conditions, suggesting that some cluster boundaries may remain sensitive to sampling variability. Such findings are not uncommon in data-driven subtype studies conducted in modestly sized clinical samples and likely reflect partial overlap between adjacent symptom profiles rather than complete separation of latent groups. Accordingly, the identified clusters should be interpreted as reproducible phenotypic tendencies within the present cohort that warrant replication in independent samples before being considered generalizable neuropsychiatric subtypes.

These observations are broadly consistent with a growing body of data-driven work addressing the heterogeneity of non-motor and neuropsychiatric manifestations in PD. Prior cluster-analytic studies have consistently identified a low-burden or normative subgroup alongside one or more symptomatic profiles, although the number and composition of derived subtypes vary across cohorts depending on the assessment battery, sample characteristics, and clustering methodology employed (Fereshtehnejad et al., 2015; Lawton et al., 2015; Mu et al., 2017). Low-burden and more symptomatic profiles have tended to emerge relatively consistently across studies, whereas the intervening profiles have varied more in their clinical composition. The present solution, comprising a recognizable low-symptom group alongside more differentiated affective-impulsive profiles, is in keeping with this literature, while the explicit inclusion of bipolar-spectrum features represents a relatively underexplored dimension that has rarely been modeled jointly with internalizing symptoms and impulsive-compulsive behaviors. The recognized challenges of unsupervised PD subtyping in samples of this size, including constraints on reproducibility and external validity across cohorts, apply equally to the present analysis (Mestre et al., 2021), reinforcing that the current profiles are best regarded as one plausible and parsimonious representation of neuropsychiatric heterogeneity rather than a definitive taxonomy.

The four-cluster solution differentiated a low-symptom group from three more symptomatic profiles: a moderate internalizing profile, a bipolar-impulsive profile, and a severe internalizing-impulsive profile, presenting differential patterns of affective dysregulation and impulsivity. Notably, bipolar-spectrum impulsive presentations and depressive-anxious impulsive presentations emerged as separate clusters, suggesting that impulsive-compulsive behaviors in Parkinson’s disease may arise through partially distinct affective and motivational pathways rather than a single homogeneous mechanism. In addition, beyond the severe internalizing-impulsive subgroup characterized by advanced disease stage and higher dopaminergic treatment burden, a separate depressive profile was identified consisting predominantly of older female patients with relatively lower lifetime psychiatric comorbidity and without elevated overall dopaminergic medication use. This finding may indicate the presence of a distinct late-life depressive phenotype in PD that is less strongly linked to pre-existing psychiatric vulnerability or disease stage. Importantly, cognitive measures did not differ significantly across clusters, and general antiparkinsonian medication load showed limited discriminatory value between profiles. Instead, the most prominent pharmacological distinction between clusters was related to amantadine exposure, suggesting a potentially specific relationship between amantadine use and affective-impulsive symptom dimensions in PD.

Importantly, covariate-adjusted analyses indicated that the identified profiles were not fully explained by demographic or disease-related differences. Although female sex was independently associated with the Moderate Internalizing profile and younger age with the Bipolar-Impulsive profile, these variables explained only a modest proportion of the variance in cluster membership. Higher dopaminergic treatment burden (total LEDD) was modestly associated with membership in the Severe Internalizing-Impulsive profile, whereas motor symptom severity showed a trend-level association. Nevertheless, the overall pattern of findings suggests that the identified clusters capture meaningful neuropsychiatric variation beyond demographic characteristics and disease-related factors alone. In this person-centered design, age and sex are not treated as classical confounders but rather as characteristics that may contribute to profile membership; covariate-adjusted models are therefore intended to complement, rather than replace, the descriptive cluster comparisons.

Cluster 2, which is the low symptom profile group, consisted of 44.3% of the sample. In one study, patients who did not report any psychiatric symptoms above thresholds was also 35.5% (Kulisevsky et al., 2008). Similar percentages are also reported for untreated PD cases (Szatmari et al., 2017). Cluster 1 and Cluster 2 represented two qualitatively distinct non-motor profiles at the lower end of symptom burden. Cluster 2, characterized by consistently low levels across all clinical dimensions, may reflect a relatively resilient subgroup of PD patients in whom neuropsychiatric symptom burden remains minimal despite active disease. In contrast, Cluster 1 exhibited moderate depressive and anxiety symptoms alongside mildly elevated anhedonia, with low ICBs, suggesting a primarily internalizing profile without prominent impulsivity, consistent with prior work highlighting depression and anxiety as core neuropsychiatric features in Parkinson’s disease (Gatto and Aldinio, 2019; Ceravolo et al., 2016). This cluster, consisted predominantly of older female patients, supporting evidence that depression in PD disproportionately affects women and may emerge independently of dopaminergic-related impulsivity mechanisms (Balestrino and Martinez-Martin, 2017; Ceravolo et al., 2009; Cao et al., 2022; Weintraub et al., 2010). Also, it suggests that not all patients are equally vulnerable to affective dysregulation or impulsive-compulsive behaviors (Fedosova et al., 2021).

Depressive groups were 46% in total and bipolar spectrum group was 16.7% of the sample. High prevalence of depressive symptoms in PD, have been reported across large cross-sectional and longitudinal studies (Molde et al., 2018; Weintraub et al., 2022). The bipolar-impulsive and severe internalizing-impulsive profiles both showed elevated ICBs but differed substantially in affective characteristics. The younger age observed in the bipolar-impulsive cluster aligns with previous findings demonstrating that both younger current age and earlier disease onset are associated with increased vulnerability to impulsive-compulsive behaviors in Parkinson’s disease (Cao et al., 2022; Weintraub et al., 2010). Emerging evidence suggests a bidirectional relationship between bipolar disorder (BD) and Parkinson’s disease (PD), potentially reflecting shared dopaminergic and neurodegenerative mechanisms, although the mechanisms underlying this association remain incompletely understood. Patients with BD demonstrate an increased long-term risk of developing PD (Bacciardi et al., 2022), with elevated risk observable decades before PD onset (Faustino et al., 2020; Jaakkola et al., 2026; Xu et al., 2024). Bipolar-spectrum features in PD have been associated with earlier disease onset, greater impulsive-compulsive behaviors, and more severe neuropsychiatric complications (Bacciardi et al., 2022; Onofrj et al., 2021). On the other hand, this cluster analysis shows that patients with bipolar spectrum features represent only a small portion of PD, even though real prevalence needs to be assessed with structured clinical interviews. Some authors have suggested that BD and PD may exhibit overlapping heterogenous neurobiological features as increased inflammation, mitochondrial dysfunction that may alter dopaminergic transmission (Ogłodek et al., 2026).

The clusters defined in this study may be related to different underlying biological processes that contribute to different psychiatric phenotypes. Cluster 4 combined severe depressive symptoms, anxiety, anhedonia, and elevated ICBs, whereas the bipolar-impulsive cluster was characterized predominantly by bipolar-spectrum features, affective instability, and impulsivity. This dissociation raises the possibility that ICBs in Parkinson’s disease may arise through at least two partially distinct affective pathways: one associated with mood instability and reward-driven impulsivity, and another embedded within a broader negative affective context. The bipolar-impulsive profile may reflect reward-related and affective-regulatory processes that partly overlap with those implicated in bipolar disorder (Ceravolo et al., 2009; Gatto and Aldinio, 2019; Grall-Bronnec et al., 2018; Zhang et al., 2021); however, the present data do not directly address these mechanisms.

In contrast, the severe internalizing-impulsive profile demonstrated the highest levels of depression, anxiety, and anhedonia. This pattern may be consistent with alterations in hedonic processing and reward sensitivity, processes that have previously been implicated in ICB vulnerability in PD (Houeto et al., 2016; Pettorruso et al., 2014); however, the present data do not directly address these mechanisms.

On the other hand, high mean scores across multiple psychiatric dimensions within a cluster do not necessarily indicate that all individuals in that subgroup exhibit uniformly elevated levels on each measure. Rather, the clustering solution reflects a clinically meaningful aggregation of individuals showing relatively greater symptom burden and shared patterns of psychopathology across these domains at the group level. The co-clustering of high anhedonia and elevated ICBs in the severe internalizing-impulsive profile is particularly striking, as these dimensions are often conceptualized as opposing motivational poles; their co-occurrence under conditions of severe neuropsychiatric burden has been previously documented in early PD (Maggi et al., 2021; Scott et al., 2024). This pattern is further consistent with prior evidence suggesting that affective symptoms, particularly anxiety and mood disturbances, contribute independently to ICBs vulnerability (Ceravolo et al., 2009; Di Rosa et al., 2022). This association has also been demonstrated in Turkish PD cohorts, where greater depressive symptom severity was independently associated with the presence of impulse-control disorders (Erkoc Ataoglu et al., 2024). Consistent with this, in another Turkish PD sample, depression and a history of impulse-control behaviors were among the predictors of suicidal ideation, underscoring the clinical significance of co-occurring affective and impulsive burden (Ozdilek and Gultekin, 2014). Previous studies have proposed that dysfunction within mesocorticolimbic and fronto-striatal networks may contribute to reward-processing abnormalities, inhibitory control deficits, and decision-making alterations associated with ICBs (Edelstyn et al., 2023; Gatto and Aldinio, 2019; Jellinger, 2025; Kimura et al., 2023).

Contrary to these factors, clusters in this study were not different for cognitive test scores. Rather the duration of PD, FAB and SMMSE scores, PD motor symptom severity and stage affected clusters differently. The most pronounced differences in disease stage and motor symptom severity were observed in Cluster 4, in particular, more affected clusters exhibited higher motor severity and more advanced disease stage, with Cluster 4 showing the highest values across these indices, suggesting increased symptom burden with disease progression, a higher prevalence of neuropsychiatric symptoms and greater non-motor burden (Balestrino and Martinez-Martin, 2017; Ceravolo et al., 2009). This group also presented with higher total LEDD scores and ICBs, consistent with prior evidence associating higher cumulative dopaminergic exposure with ICB vulnerability (Gatto and Aldinio, 2019, Molde et al., 2018; Binck et al., 2020; Marković et al., 2020; Zhang et al., 2021). Notably, clusters differed in total LEDD rather than dopamine agonist- or levodopa-specific doses, supporting the view that overall medication burden may be more relevant than individual agents (Contin et al., 2023; Grall-Bronnec et al., 2018; Staubo et al., 2024). Although higher amantadine load was observed in more symptomatic clusters, this may reflect greater clinical complexity and advanced treatment needs rather than a direct causal role in ICB expression (Vitale et al., 2019). Literature about the non-motor symptom effects of amantadine is contradictory as there are reports both for elevated mood but also increased symptom burden (Vanle et al., 2018; Krzystanek and Pałasz, 2020; Sodré et al., 2010). However, Cluster 4 consisted of only 12 patients (9.5% of the sample), and therefore the observed findings should be interpreted cautiously, as severe neuropsychiatric presentations may have been influenced by selection or participation bias, with more clinically impaired individuals potentially being either overrepresented or less likely to participate consistently in research assessments. In addition to neurobiological and treatment-related mechanisms, the greater psychiatric burden observed in more advanced disease stages may also reflect increasing psychosocial stress associated with PD progression. Progressive physical disability, reduced autonomy, social withdrawal, caregiver dependence, and functional impairment may contribute substantially to depressive and anxiety symptoms over time (Sagna et al., 2014). Therefore, the observed association between disease severity and neuropsychiatric burden likely reflects a complex interaction between neurodegenerative, pharmacological, and psychosocial factors (McDaniels et al., 2023).

### Clinical implications

Clinically, these findings highlight the substantial heterogeneity of non-motor symptom profiles in Parkinson’s disease and support the need for transdiagnostic, person-centered assessment approaches rather than evaluating psychiatric symptoms in isolation. Distinct clusters characterized by differing combinations of depressive symptoms, anxiety, anhedonia, bipolar-spectrum features, and impulsivity may reflect differing underlying processes and clinical needs. Recognizing these profiles may facilitate earlier identification of vulnerable patients, improve individualized treatment planning, and encourage more integrated multidisciplinary management strategies targeting both neurological and neuropsychiatric dimensions of Parkinson’s disease, in addition to better characterizing the neurobiological pathways involved beyond cognitive measures.

Practically, these profiles may inform how neuropsychiatric symptoms are screened and managed in PD clinics. Rather than assessing depression, anxiety, or impulse-control behaviors in isolation, the co-occurrence patterns observed here suggest that a positive screen in one domain should prompt structured evaluation of the others, for example, identifying bipolar-spectrum features or impulsivity in a patient presenting primarily with mood symptoms, or screening for affective burden in patients flagged for impulse-control behaviors. The Bipolar-Impulsive profile, characterized by younger age and elevated impulsivity, may warrant particular attention to impulse-control behaviors, especially in patients receiving dopaminergic therapies. Conversely, the Severe Internalizing-Impulsive profile, characterized by the highest overall neuropsychiatric burden and greater clinical complexity, may benefit from closer psychiatric monitoring as part of routine follow-up. In multidisciplinary settings, recognizing these profiles could support more targeted referral between neurology and psychiatry, with brief transdiagnostic screening instruments helping neurologists identify patients who would benefit from psychiatric assessment and helping psychiatrists contextualize affective and impulsive symptoms within the broader clinical presentation of Parkinson’s disease. Such profile-based stratification remains a clinical hypothesis that requires prospective validation before it can guide routine practice.

This study has several important strengths. First, it adopted a multidimensional transdiagnostic approach by simultaneously evaluating depression, anxiety, anhedonia, bipolar-spectrum features, and impulsive-compulsive behaviors in Parkinson’s disease. Second, the sample size was supported by power analysis and data were collected from two independent university centers, increasing generalizability. Third, cluster validity and stability were rigorously evaluated using multiple complementary methods, including Silhouette coefficient, Davies-Bouldin Index, LDA-based separability, bootstrap resampling, Adjusted Rand Index, and Jaccard similarity analyses. Finally, the identified clusters were not only statistically robust but also clinically interpretable and consistent with real-world clinical observations and disease characteristics.

Several limitations should also be considered. First, the cross-sectional design precludes causal or longitudinal interpretations, and the identified clusters should therefore be viewed as patterns of co-occurring symptoms rather than stable trajectories. The temporal stability and prognostic significance of these profiles remain unknown, and longitudinal studies are needed to determine whether they persist over time or predict future neuropsychiatric outcomes. Second, clustering solutions are inherently sample- and variable-dependent, and unsupervised subtype discovery in samples of this size may be susceptible to limitations in reproducibility and external validity across cohorts (Mestre et al., 2021). Furthermore, the exclusive use of k-means clustering may limit methodological robustness, as subtype solutions can be sensitive to the choice of clustering algorithm. Replication in larger, independent cohorts with harmonized assessment batteries, as well as application of alternative approaches such as latent profile analysis, is therefore warranted before these profiles can be considered generalizable. Third, the four clusters differed substantially in size, with the Severe Internalizing-Impulsive profile comprising only 12 participants (9.5%). Although stability analyses supported the reproducibility of the overall solution, unequal and small cluster sizes may reduce the precision of between-cluster comparisons and limit the generalizability of the smallest profile, which should be regarded as preliminary pending replication. Consistent with this, in the covariate-adjusted analysis the marginal association of motor severity with this cluster may reflect limited statistical power and warrant confirmation in larger samples. Fourth, the selected psychometric and clinical variables may not fully capture the complexity of neuropsychiatric phenotypes in Parkinson’s disease, as sleep problems and psychotic symptoms have not been assessed. The absence of neurobiological measures and the use of a single general anhedonia scale further limited mechanistic interpretation. In addition, reliance on self-report measures may introduce reporting bias. Longitudinal and multimodal replication studies are needed to confirm these findings.

## Conclusion

In this outpatient group of PD with no dementia diagnosis, findings support a multidimensional model in which non-motor symptoms in PD accumulate in four clusters across affective symptoms, impulsivity, disease severity, and individual vulnerability. Recognizing this heterogeneity may facilitate more precise assessment and more personalized management strategies in patients with Parkinson’s disease.

## Data Availability

The datasets presented in this article are not readily available because the generated dataset is available upon reasonable request and IRB approval. Requests to access the datasets should be directed to hyapici@ku.edu.tr.
