# Supplementary material for: A transdiagnostic approach to non-motor symptoms in Parkinson’s disease: exploring internalizing, bipolar spectrum, and impulsive compulsive behavior profiles via cluster analysis
Source: Front Aging Neurosci. 2026 Aug 25;18:1898498. doi: 10.3389/fnagi.2026.1898498 (PMC13550225; doi:10.3389/fnagi.2026.1898498)
Supplement: Supplementary file 1 [file Data_Sheet_1.docx]

**Title: A Transdiagnostic Approach to Non-Motor Symptoms in Parkinson’s Disease: Exploring Internalizing, Bipolar Spectrum, and Impulsive Compulsive Behavior Profiles via Cluster Analysis**

Ardıl Bayram Şahin, Mahmoud Mahmoudi, Yasemin Kadandır, İbrahim Melih Öztürkmen**,** Elif Sude Özaşik, [Roza Gök](https://pubmed.ncbi.nlm.nih.gov/?sort=pubdate&term=G%C3%B6k+R&cauthor_id=40827341), Muhammet Nesip Seyidoğlu, Çınar Öztosun**,** Özgür Öztop Çakmak, Gül Yalçın Çakmaklı, Fahriye Feriha Özer , Hale Yapici Eser

**Corresponding author:**

Hale Yapıcı Eser, MD PHD

Associate Professor of Psychiatry

Koç University School of Medicine, İstanbul, Türkiye

Koç University Graduate School of Health Sciences, Neuroscience Program, İstanbul, Türkiye

Koç University Research Center for Translational Medicine, İstanbul, Türkiye

hyapici@ku.edu.tr

**Table S1A Severity of Impulsive-Compulsive Behaviors and QUIP-RS Load**

| **Variable** | **Mean ± SD** |
| --- | --- |
| **ICB Subtypes** |  |
| Pathological Gambling | 0.31 ± 1.37 |
| Hypersexuality | 1.48 ± 2.87 |
| Compulsive Shopping | 1.70 ± 2.74 |
| Compulsive Eating | 2.37 ± 3.34 |
| Hobbyism | 2.32 ± 2.96 |
| Punding | 2.00 ± 2.62 |
| Dopamine Dysregulation Syndrome (DDS) | 2.10 ± 3.47 |
| **Overall** |  |
| ICB Load | 1.39 ± 1.20 |

ICB load is defined as the total number of ICB subtypes that exceed the cut-off scores QUIP-RS (Probst et al. 2014). *QUIP-RS* Questionnaire for Impulsive-Compulsive Disorders in Parkinson’s Disease–Rating Scale, *ICB* Impulsive-Compulsive Behavior, *SD* Standard Deviation

**Table S1B Prevalence of Impulsive-Compulsive Behaviors**

| **Variable** | **n (%)** |
| --- | --- |
| **ICB Subtypes** |  |
| Pathological Gambling | 4 (3.2) |
| Hypersexuality | 13 (10.3) |
| Compulsive Shopping | 17 (13.5) |
| Compulsive Eating | 33 (26.2) |
| Hobbyism | 30 (23.8) |
| Punding | 41 (32.5) |
| Dopamine Dysregulation Syndrome (DDS) | 37 (29.4) |
| **Overall** |  |
| Any ICBs | 58 (46.0) |

Prevalence values are based on QUIP-RS cut-off scores (Probst et al. 2014). *QUIP-RS* Questionnaire for Impulsive-Compulsive Disorders in Parkinson’s Disease–Rating Scale*, ICB* Impulsive-Compulsive Behavior, *SD* Standard Deviation

**Table S1C. Distribution of Impulsive-Compulsive Behaviors per Patient**

| **Number of ICB** | **n (%)** |
| --- | --- |
| 0 | 68 (54) |
| 1 | 19 (15) |
| 2 | 8 (6.4) |
| 3 | 9 (7.1) |
| 4 | 5 (4.0) |
| 5 | 9 (7.1) |
| 6 | 8 (6.4) |

Prevalence values are based on QUIP-RS cut-off scores (Probst et al. 2014). *ICB* Impulsive-Compulsive Behavior

**Fig. S1** Radar plot depicting standardized mean levels (z-scores) across key domains for the six-cluster (k = 6) k-means solution. Variables include depression (PHQ-9), anxiety (GAD-7), anhedonia (SHAPS), bipolar spectrum features (MDQ), and impulse–compulsive behaviors (QUIP-RS), enabling comparison across clusters. Although the six-cluster solution was supported by quantitative indices during model evaluation, visual inspection indicates substantial overlap in profile geometry, with clusters differing mainly in magnitude rather than qualitative pattern. This fragmentation without meaningful interpretive gain contrasts with the clearer, more distinct configurations observed in the four-cluster solution (Figure 3 in main text), supporting the selection of the more parsimonious four-cluster model as the final solution.

**Fig. S2** Mean pairwise Jaccard similarity across repeated k-means runs (n_init = 1) for candidate cluster solutions (k = 2–8). Values are presented as mean ± 1 standard deviation across iterations. Stability decreased progressively with increasing k, consistent with greater fragmentation and heightened sensitivity to initialization at higher cluster counts. Mid-range solutions, particularly k = 4, demonstrated moderate stability.

**Fig. S3.** Mean pairwise Jaccard similarity across repeated runs with extensive internal optimization (n_init = 100). Under this configuration, solutions up to k = 6 exhibited near-perfect convergence, indicating consistent recovery of highly similar partitions across iterations. Stability declined beyond k = 4, suggesting reduced robustness for higher-order cluster solutions.

**Table S2 Comparisons of Depression, Anxiety, Anhedonia, Mood Instability and ICBs Across the Six Cluster Solution**

|  | **Cluster 1 (n=10)** | **Cluster 2 (n=54)** | **Cluster 3 (n=15)** | **Cluster 4 (n=4)** | **Cluster 5 (n=28)** | **Cluster 6 (n=15)** | **Kruskal–Wallis Test** | | | **Dunn’s Test** | |
| --- | --- | --- | --- | --- | --- | --- | --- | --- | --- | --- | --- |
| **Variable** | **Mean ± SD** | **Mean ± SD** | **Mean ± SD** | **Mean ± SD** | **Mean ± SD** | **Mean ± SD** | **Value** | **df** | ***P*** | **Pairwise comparisons** | **Adj. Sig** |
| PHQ-9 | 14.10 ± 3.28 | 4.04 ± 1.80 | 8.60 ± 3.29 | 14.25 ± 5.68 | 9.29 ± 2.83 | 8.60 ± 4.22 | 74.266 | 5 | **<0.001**** | Cluster 1- Cluster 2  Cluster 1- Cluster 3  Cluster 1- Cluster 4  Cluster 1- Cluster 5  Cluster 1- Cluster 6  Cluster 2- Cluster 3  Cluster 2- Cluster 4  Cluster 2- Cluster 5  Cluster 2- Cluster 6  Cluster 3- Cluster 4  Cluster 3- Cluster 5  Cluster 3- Cluster 6  Cluster 4- Cluster 5  Cluster 4- Cluster 6  Cluster 5- Cluster 6 | <0.001**  0.356  1.000  0.748  0.230  <0.001**  0.002*  <0.001**  <0.001**  1.000  1.000  1.000  1.000  1.000  1.000 |
| GAD-7 | 14.50 ± 3.10 | 2.04 ± 1.61 | 5.27 ± 2.40 | 11.25 ± 5.74 | 5.54 ± 3.07 | 8.07 ± 3.99 | 72.622 | 5 | **<0.001**** | Cluster 1- Cluster 2  Cluster 1- Cluster 3  Cluster 1- Cluster 4  Cluster 1- Cluster 5  Cluster 1- Cluster 6  Cluster 2- Cluster 3  Cluster 2- Cluster 4  Cluster 2- Cluster 5  Cluster 2- Cluster 6  Cluster 3- Cluster 4  Cluster 3- Cluster 5  Cluster 3- Cluster 6  Cluster 4- Cluster 5  Cluster 4- Cluster 6  Cluster 5- Cluster 6 | <0.001**  0.036*  1.000  0.013*  1.000  0.005*  0.003*  <0.001**  <0.001**  1.000  1.000  1.000  1.000  1.000 |
| SHAPS | 1.80 ± 1.48 | 0.72 ± 1.05 | 0.73 ± 1.10 | 8.00 ± 2.16 | 1.79 ± 1.48 | 1.00 ± 1.36 | 29.086 | 5 | **<0.001**** | Cluster 1- Cluster 2  Cluster 1- Cluster 3  Cluster 1- Cluster 4  Cluster 1- Cluster 5  Cluster 1- Cluster 6  Cluster 2- Cluster 3  Cluster 2- Cluster 4  Cluster 2- Cluster 5  Cluster 2- Cluster 6  Cluster 3- Cluster 4  Cluster 3- Cluster 5  Cluster 3- Cluster 6  Cluster 4- Cluster 5  Cluster 4- Cluster 6  Cluster 5- Cluster 6 | 0.288  0.549  0.479  1.000  1.000  1.000  <0.001**  0.009*  1.000  0.002*  0.125  1.000  0.252  0.009*  0.610 |
| MDQ | 3.70 ± 1.83 | 1.00 ± 1.33 | 6.27 ± 2.09 | 4.00 ± 2.16 | 1.46 ± 1.07 | 7.33 ± 2.23 | 80.418 | 5 | **<0.001**** | Cluster 1- Cluster 2  Cluster 1- Cluster 3  Cluster 1- Cluster 4  Cluster 1- Cluster 5  Cluster 1- Cluster 6  Cluster 2- Cluster 3  Cluster 2- Cluster 4  Cluster 2- Cluster 5  Cluster 2- Cluster 6  Cluster 3- Cluster 4  Cluster 3- Cluster 5  Cluster 3- Cluster 6  Cluster 4- Cluster 5  Cluster 4- Cluster 6  Cluster 5- Cluster 6 | 0.003*  1.000  1.000  0.189  1.000  <0.001**  0.192  1.000  <0.001**  1.000  <0.001**  1.000  1.000  1.000  <0.001** |
| QUIP-RS | 2.20 ± 1.99 | 0.28 ± 0.76 | 1.27 ± 0.96 | 4.00 ± 1.83 | 0.82 ± 1.25 | 5.33 ± 0.72 | 71.142 | 5 | **<0.001**** | Cluster 1- Cluster 2  Cluster 1- Cluster 3  Cluster 1- Cluster 4  Cluster 1- Cluster 5  Cluster 1- Cluster 6  Cluster 2- Cluster 3  Cluster 2- Cluster 4  Cluster 2- Cluster 5  Cluster 2- Cluster 6  Cluster 3- Cluster 4  Cluster 3- Cluster 5  Cluster 3- Cluster 6  Cluster 4- Cluster 5  Cluster 4- Cluster 6  Cluster 5- Cluster 6 | 0.020*  1.000  1.000  0.815  0.099  0.016*  0.003*  1.000  <0.001**  1.000  1.000  0.008*  0.068  1.000  <0.001** |

Values are presented as test statistics from Kruskal–Wallis analyses. Pairwise comparisons were performed using Dunn–Bonferroni post hoc tests when the overall test was significant. Adjusted p-values (Adj. Sig.) reflect Bonferroni correction for multiple comparisons. *PHQ-9* Patient Health Questionnaire-9, *GAD-7* General Anxiety Disorder-7, *SHAPS* Snaith-Hamilton Pleasure Scale, *MDQ* Mood Disorder Questionnaire, *QUIP-RS* Questionnaire for Impulsive-Compulsive Disorders in Parkinson’s Disease–Rating Scale, *SD* Standard Deviation, **p* < 0.05 ***p* < 0.001

**Table S3 Treatment Characteristics Across the Four Cluster Solution**

| **Variable** | **Cluster 1 (n=37)**  **n (%)** | **Cluster 2 (n=56)**  **n (%)** | **Cluster 3 (n=21)**  **n (%)** | **Cluster 4 (n=12)**  **n (%)** | ***P*** |
| --- | --- | --- | --- | --- | --- |
| Treatment Usage | 35 (94.6) | 51 (91.1) | 21(100) | 11 (91.7) | 0.535 |
| Levodopa Usage | 28 (75.7) | 40 (71.4) | 15 (71.4) | 11 (91.7) | 0.517 |
| DA Usage | 20 (54.1) | 30 (53.6) | 14(66.7) | 6 (50) | 0.724 |
| MAO-I Usage | 14 (37.8) | 18 (32.1) | 7 (33.3) | 2 (16.7) | 0.602 |
| Amantadine Usage | 5 (13.5) | 6 (10.7) | 8 (38.1) | 6 (50) | 0.002* |

Pairwise comparisons were performed using Chi-Square test with Bonferroni correction (**p* < 0.01).

*SD* Standard Deviation. *DA* Dopamine Agonist, *MAO-I* Monoamine oxidase inhibitor
